# Intrinsic and selected resistance to antibiotics binding the ribosome: analyses of *Brucella *23S *rrn*, L4, L22, EF-Tu1, EF-Tu2, efflux and phylogenetic implications

**DOI:** 10.1186/1471-2180-6-84

**Published:** 2006-10-02

**Authors:** Shirley M Halling, Allen E Jensen

**Affiliations:** 1 Bacterial Diseases of Livestock Research Unit, National Animal Disease Center, Agricultural Research Service, United States Department of Agriculture, 2300 Dayton Avenue, Ames, IA 50010, USA

## Abstract

**Background:**

*Brucella *spp. are highly similar, having identical 16S RNA. However, they have important phenotypic differences such as differential susceptibility to antibiotics binding the ribosome. Neither the differential susceptibility nor its basis has been rigorously studied. Differences found among other conserved ribosomal loci could further define the relationships among the classical *Brucella *spp.

**Results:**

Minimum inhibitory concentration (MIC) values of *Brucella *reference strains and three marine isolates to antibiotics binding the ribosome ranged from 0.032 to >256 μg/ml for the macrolides erythromycin, clarithromycin, and azithromycin and 2 to >256 μg/ml for the lincosamide, clindamycin. Though sequence polymorphisms were identified among ribosome associated loci 23S *rrn*, *rpl*V, *tuf*-1 and *tuf*-2 but not *rplD*, they did not correlate with antibiotic resistance phenotypes. When spontaneous erythromycin resistant (ery^R^) mutants were examined, mutation of the peptidyl transferase center (A2058G Ec) correlated with increased resistance to both erythromycin and clindamycin. *Brucella *efflux was examined as an alternative antibiotic resistance mechanism by use of the inhibitor L-phenylalanine-L-arginine β-naphthylamide (PAβN). Erythromycin MIC values of reference and all ery^R ^strains, except the *B. suis *ery^R ^mutants, were lowered variably by PAβN. A phylogenetic tree based on concatenated ribosomal associated loci supported separate evolutionary paths for *B. abortus, B. melitensis*, and *B. suis/B. canis*, clustering marine *Brucella *and *B. neotomae *with *B. melitensis*. Though *Brucella ovis *was clustered with *B. abortus*, the bootstrap value was low.

**Conclusion:**

Polymorphisms among ribosomal loci from the reference *Brucella *do not correlate with their highly differential susceptibility to erythromycin. Efflux plays an important role in *Brucella *sensitivity to erythromycin. Polymorphisms identified among ribosome associated loci construct a robust phylogenetic tree supporting classical *Brucella *spp. designations.

## Background

Brucellosis is a zoonotic disease caused by the Gram-negative bacterium *Brucella*. It is taxonomically related to plant pathogens and other animal symbionts and is transmitted to humans from infected domestic animals and wildlife through contact during animal husbandry practices, meat production, or by ingestion of unpasteurized milk products. The genus *Brucella *contains six classical species reflecting host preferences [1,2], and additional species have been proposed to include marine isolates from seal, dolphin, and porpoise [3]. The classical species and their hosts are: *B. abortus*, bovine; *B. melitensis*, caprine; *B. suis*, porcine; *B. ovis*, ovine; *B. canis*, canine; and *B. neotomae*, desert wood rat. However, *B. suis and B. canis *have similar metabolic profiles [4] and genomic maps [5], supporting their close relationship. Similarly, the metabolic characteristics and phage susceptibility of *B. suis *biovar 5 are more like that of *B. melitensis *rather than *B. suis *[6,7].

The classical *Brucella *spp. designations are still widely used to emphasize important pathogenicity, virulence, and host preference differences among the *Brucella *even though similarity among the ribosomal RNA loci led to the designation of *Brucella *as a monospecific species [8,9], *B. melitensis*. *Brucella *speciation may have arisen as a result of their isolation due to different preferred hosts and to divergence of the host species [10] even though their 16S *rrn *loci are identical [11-14]. In any case, discordant genotype/phenotype may require the use of other widely conserved loci to define bacterial species [15,16].

Meyer [17] found differences in sensitivity to erythromycin among the classical species of *Brucella *and their biovars by measuring inhibition of growth using high and low concentration antibiotic discs. *Brucella abortus *biovars except biovar 2 were resistant to erythromycin, and *B. ovis*, *B. melitensis*, and *B. canis *were intermediate in resistance between *B. abortus *and *B. suis*. Only *B. suis *strains were sensitive to the high concentration antibiotic discs. Meyer argued that investigating the ribosomal structure could explain these differences in sensitivity and generate critical knowledge "to account for and recapitulate the lineage of species and biotypes of *Brucella*".

Bacterial susceptibility to macrolide and lincosamide antibiotics results from their binding to 23S rRNA, inhibiting protein synthesis by blocking the peptide exit tunnel [18-20]. Bacteria can become resistant to macrolides and lincosamides by spontaneous mutations of ribosomal associated loci or by increased efflux. Resistance to macrolides and lincosamides is commonly due to (i) mutation of 23S *rrn *causing a reduction in the binding of the antibiotics to the peptidyl transferase center [21,22], typically nts A2058, A2059, A2062, and C2611, *Escherichia coli *(Ec) 23S rRNA numbering, (ii) mutation of ribosomal proteins L4 or L22 leading to widening the entrance to the peptide exit tunnel allowing access to the tunnel even in the presence of the antibiotics [18,20,23-25], (iii) methylation of ribosomal 23S rRNA [26], or (iv) increased efflux [27,28]. Bacterial resistance to synthetic macrolides or ketolides can be conferred by mutation of ribosome associated factor EF-Tu [29].

There are several families of efflux pumps, though few non-RND (resistance nodulation division) family efflux pumps cause intrinsic or spontaneous resistance of Gram-negative bacteria to common clinical antibiotics [27,28,30,31]. Inability to demonstrate efflux activity however does not necessarily mean a lack of efflux. Efficiency of efflux of antibiotics is variable, being dependent on the structure of the antibiotic. Antibiotic resistance can be complex as observed for *Haemophilus influenzae *L22 mutant HMC-C [32]. For this mutant, an increase in macrolide MIC values was only shown in the presence of efflux [33].

Here, we show that the large differences in relative intrinsic susceptibilities of reference strains of *Brucella *and three marine isolates to macrolide antibiotics and a lincosamide do not correlate with ribosomal associated polymorphisms. We establish that antibiotic efflux plays an important role in differential antibiotic susceptibility in *Brucella*. A robust phylogenetic tree constructed from concatenation of ribosome associated polymorphisms illuminates relationships among the *Brucella*.

## Results

### MIC determination by Etest

**Figure 1:**
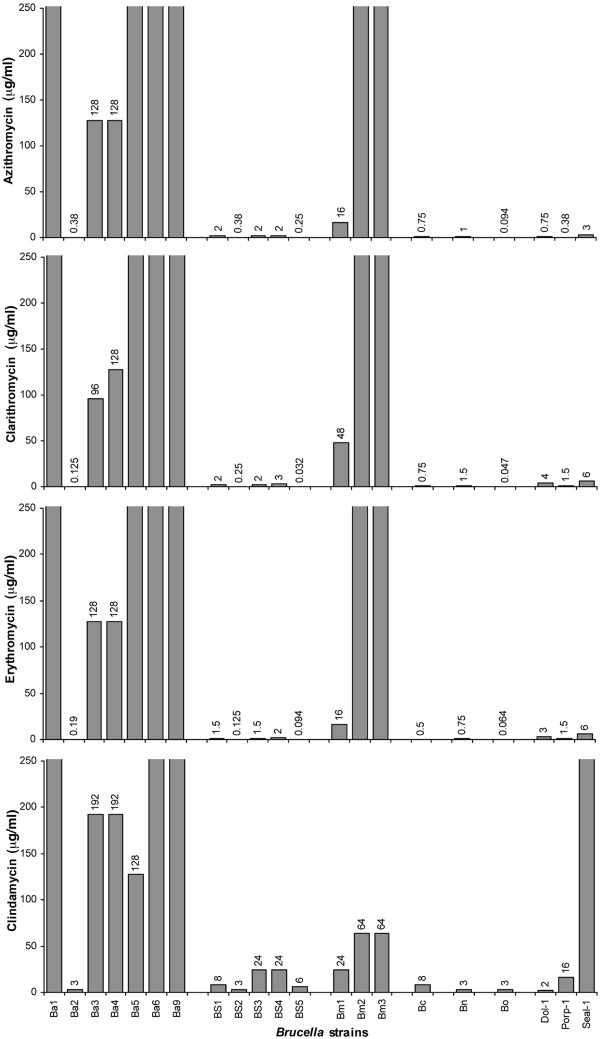
**Minimal inhibitory concentration (MIC) of antibiotics to *Brucella *reference strains and marine isolates**. MICs of three macrolides, azithromycin, clarithromycin, erythromycin, and the lincosamide, clindamycin, were determined by Etest. Maximum MIC measurable by the Etest is 256 μg/ml for each of the antibiotics. *Brucella *strains as listed in Table 1. Ba = *B. abortus *; Bs = *B. suis*; Bm = *B. melitensis*; Bc = *B. canis*; Bn = *B. neotomae*; Bo = *B. ovis*; numbers following species designate biovar.

The relative MIC values of the classical *Brucella *spp. and three marine isolates (Table 1) to macrolides and a lincosamide were determined by use of the Etest. Log-fold differences in MIC values were found (Fig. 1). The susceptibility of *Brucella *was similar for the three macrolides erythromycin, azithromycin, and clarithromycin. Only *B. abortus*, except biovar 2, and *B. melitensis *had MIC values of ≥ 16 μg/ml. The pattern of sensitivity of *Brucella *to the lincosamide clindamycin differed from that of the macrolides. Generally, MIC values were higher for clindamycin than for the macrolides. *Brucella abortus*, except biovar 2, was the most resistant to clindamycin, having MIC values of ≥ 128 ug/ml. Only *B. melitensis *biovars 2 and 3 had lower MIC values for clindamycin than for erythromycin. For *B. suis*, clindamycin MIC values ranged from a low of 3 μg/ml to a high of 24 μg/ml. The other *Brucella *with the exception of the seal isolate, ranged from 2 to 64 μg/ml. The seal isolate was resistant to clindamycin.

### 23S rrn sequence comparisons

Sequences of two regions of the *Brucella *23S *rrn *encoding 2498 nts (69 to 1678 and 1920 to 2807), including sites of 23S *rrn *mutations known to increase bacterial resistance to macrolides and clindamycin were determined and compared (Table 2). Mixtures of cells or DNA (1:3) with disparate 23S *rrn *sequences were amplified to demonstrate that heterogeneity among the three 23S *rrn *copies would be detectable (data not shown). Though the distal portion of 23S *rrn*C from the genomic *B. suis *1330 sequence could not be amplified with either of two primer pairs that were complementary to the published *B. suis *23S *rrn*C genomic sequence, amplification was successful using primers homologous to internal, conserved genomic *rrn*C sequences from all three *Brucella *genomes and sequences flanking *rrn*C from *B. abortus *and *B. melitensis*. The amplified distal portion of *rrn*C from *B. suis *1330 was identical in sequence to that of *rrn*A and *rrn*B from *B. suis*. Among the 23S *rrn *sequences from *Brucella*, three polymorphic and three monomorphic sites were identified. In addition, three monomorphisms were identified in the 23S *rrn *intervening sequences.

23S *rrn *polymorphisms were detected at nts 1085 (934, Ec), 1564 (1423, Ec), and 2632 (2610, Ec), clustering the *Brucella *into three groups: (1) *B. abortus*, (2) *B. canis *and *B. suis*, except biovar 5, and (3) *B. melitensis*, *B. ovis*, *B. neotomae*, *B. suis *biovar 5, and the dolphin, seal and porpoise isolates (Table 2). Note that all the 23S *rrn Brucella *positions are numbered based on *B. abortus *23S *rrn*A, including the intervening sequence. The only polymorphism that occurred in the peptidyl transferase center was nt 2632 (2610, Ec). No correlation could be made between the polymorphisms and relative antibiotic susceptibility. Other sites known to affect susceptibility to macrolides and clindamycin were not polymorphic.

Monomorphisms were found in both 23S *rrn *and in the 23S *rrn *intervening sequences. Monomorphisms were identified in 23S rRNAs from dolphin (A955G); *B. neotomae *(insertion of a C between nt 1002–1006); and *B. suis *biovar 5 (T2090C). Several intervening sequences of the *Brucella *23S *rrn *loci varied from the consensus sequence reported by Bricker [34]. The C indel in the intervening sequences of *B. melitensis *16 M, forming a string of six Cs instead of five beginning at nt 222, reported by Bricker was confirmed. The other two monomorphisms occurred in *B. suis *biovar 5 (C219T) and *B. melitensis *biovar 3 (C206T).

### L4 analyses

Though the GenBank *Brucella *genomic *rplD *sequences encoding ribosomal protein L4, differed due to an indel in *rplD *found only in the *B. melitensis *16 M genomic sequence, we did not observe this indel in our sequence of *rplD *from *B. melitensis *16 M. We found the three *rplD *genes from the genomes were identical. Though no polymorphisms were identified among the *rplD *sequences, three monomorphisms were discovered among their amino termini. Two sequence transitions, *B. neotomae *(G108A) and *B. suis *biovar 2 (C213T), were found. Both of which were silent. A transversion identified in the porpoise isolate (G314T) would replace an Arg, a charged amino acid (aa), with Leu, a noncharged one.

### L22 polymorphisms

**Figure 2:**
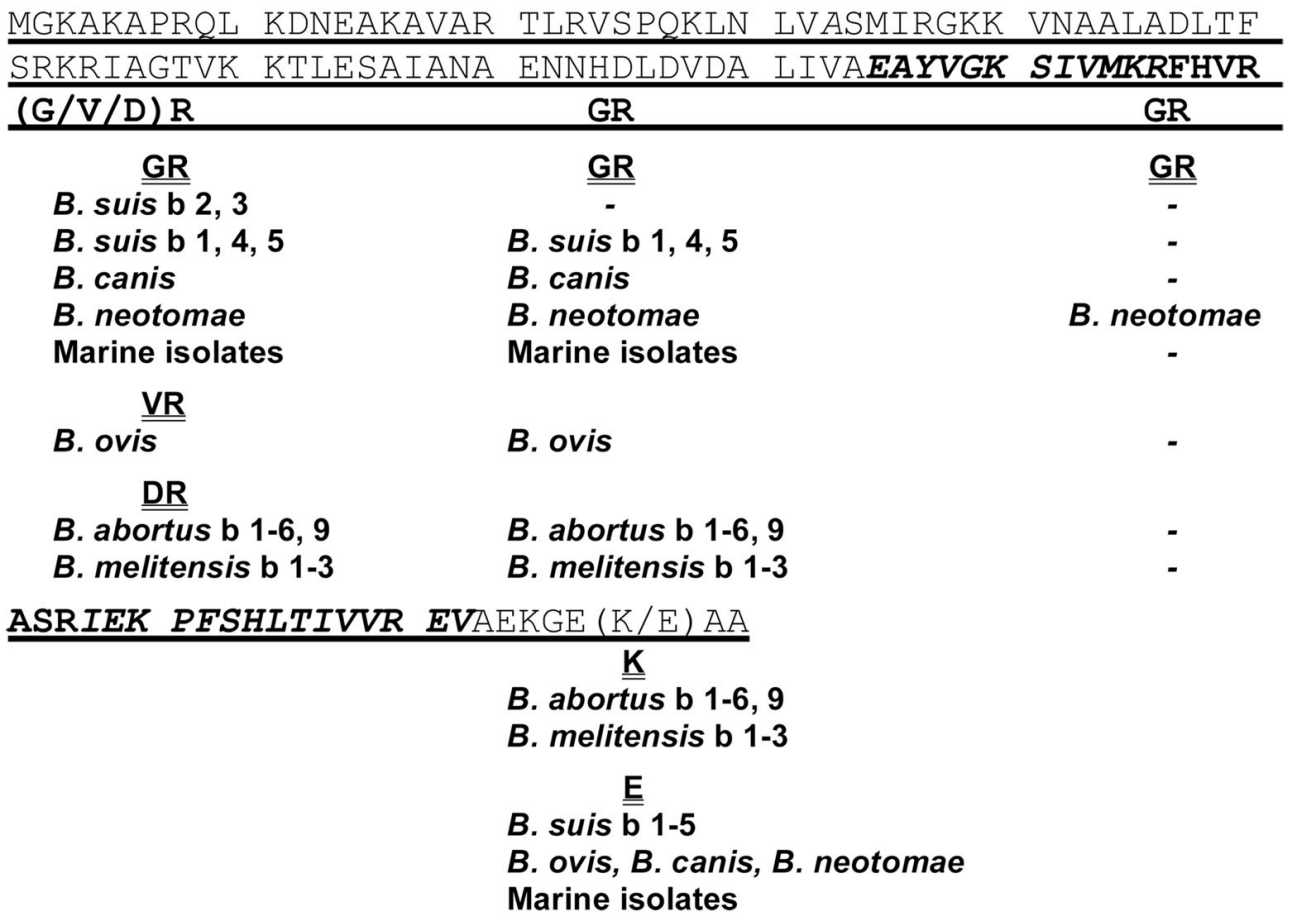
**Ribosomal protein L22 polymorphisms among *Brucella *reference strains and three marine isolates**. The putative peptide sequence of L22 is underlined, parentheses bracket polymorphic sites and list amino acids found among L22 peptides at that site. In regions where sequence was variable, the sequence for each putative *Brucella *L22 is given below. Amino acids occurring in the stalk of the β-hairpin appear in ***bold-italics ***and amino acids occurring in the loop of the β-hairpin are in **bold**. Beneath amino acids that are double underlined is a list of *Brucella *strains containing those aa. The single letter code is used to denote the putative aa sequence of the peptides; b = biovar and numbers following "b" designate biovar numbers. Accession numbers of *rplV *sequences for each strain are deposited in GenBank and are listed in Materials.

**Figure 3:**
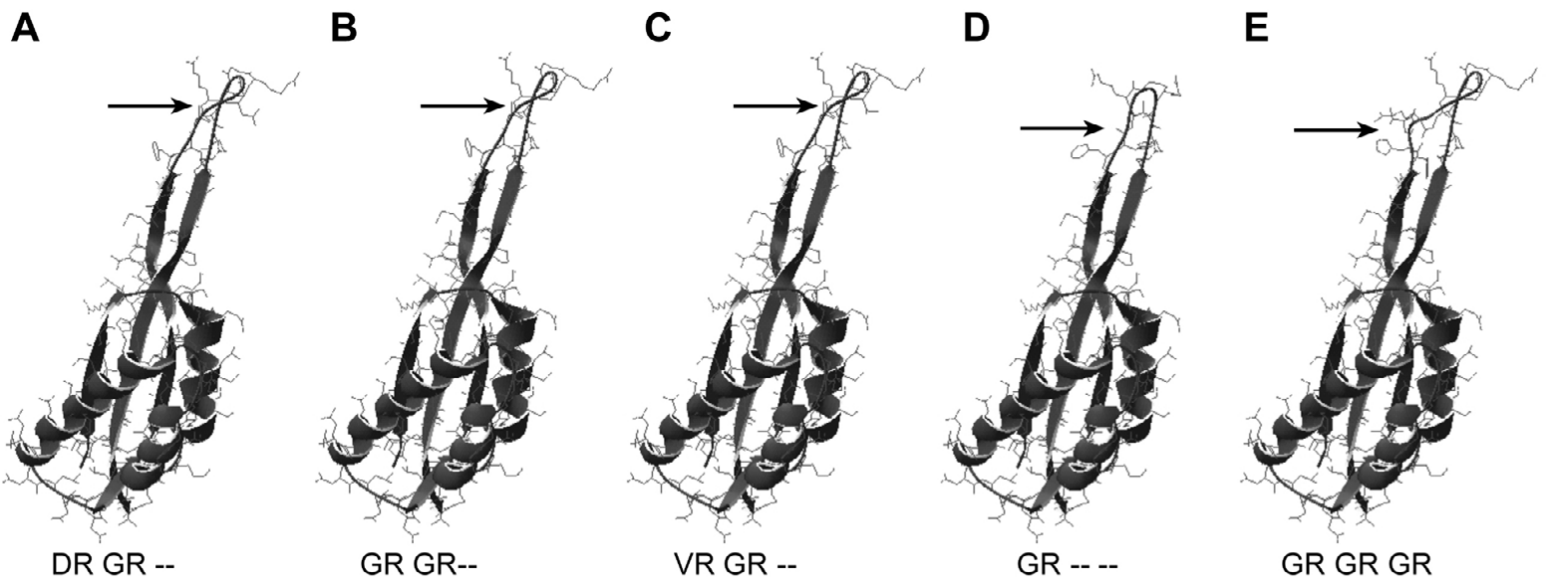
**Ribbon diagrams showing predicted secondary structures of ribosomal L22 proteins**. The divergent, putative aa sequences from the reference *Brucella *strains and three marine isolates are found in Fig. 2. The ribbon structures for each group is as follows: (A) *B. abortus *and *B. melitensis*; (B) *B. canis*, *B. suis *biovars 1, 4, and 5, and the marine isolates, (C) *B. ovis*; (D) *B. suis *biovars 2 and 3; and (E) *B. neotomae*. Structures were predicted based on coordinates of L22 from *Thermus thermophilus *[52] and prepared using Swiss-Pdb viewer [50, 51]. Region containing β-hairpin loops (→).

Putative L22 sequences from the *Brucella *reference strains and three marine isolates (Fig. 2) were determined and their tertiary structures predicted and compared by using Swiss-Pdb Viewer (Fig. 3). Among the *Brucella *putative L22 sequences, all variations except one occurred in the β-hairpin loops or near the carboxy termini. *Brucella suis *biovar 5 alone had an alternate Ala codon at aa 44. The *Brucella *β-hairpin loops were polymorphic and variable in length due to variable copy numbers of a two aa motif, Gly-Arg. The lengths of all the β-hairpin loops of putative L22 peptides except those from *B. neotomae *and *B. suis *biovars 2 and 3 were equal, 11 aa. The β-hairpin loops of L22 from *B. suis *biovars 2 and 3 were shorter due to a net two aa (Gly-Arg) deletion, while the β-hairpin loop from *B. neotomae *was longer due to a net two aa (Gly-Arg) insertion. Within the loop of the β-hair pin at aa 101, there was either a Gly, Val, or an Asp. While the variation of sequence at aa 101 of L22 did not greatly affect the predicted tertiary structures, the indels did (Fig. 3). A polymorphism was also identified very near the 3'-end of *rplV*. L22 polymorphic sites grouped the *Brucella *differently than other loci in this study. Putative L22 sequences from *B. abortus *and *B. melitensis *were identical. *Brucella suis *biovars 1, 4 and 5, and the marine isolates were identical. Putative L22 sequences from *B. suis *biovars 2 and 3 differed from those of biovars 1, 4 and 5 in having one rather than two Gly-Arg motifs. Putative L22 sequences from both *B. ovis *and *B. neotomae *were unique. No correlation could be made between relative antibiotic susceptibilities of the *Brucella *strains and their L22 sequences.

### EF-Tu sequence comparisons

Though the nt sequences of EF-Tu loci, *tuf*-1 and *tuf*-2, were polymorphic (Table 3), the putative peptide sequences of EF-Tu1 and -2 were conserved. In most *Brucella *strains, *tuf*-1 and *tuf-*2 sequences were identical. In the cases where they were not identical, they varied by a single nt near either the 5' or 3' termini of the genes, namely nt 12 and nt 1158. Unlike the 23S *rrn *sequences, the sequences of *tuf*-1 and *tuf*-2 from *B. abortus *were more similar to those from *B. suis *than from *B. melitensis*. The reference *B. abortus *biovar 1 strain, 544, differed from that of the sequenced strain, *B. abortus *biovar 1 strain 9–941 (Table 3). Nt 1158 of *tuf*-2 from *B. abortus *544 differed from *B. abortus *biovar 1 strains 2308 and 9–941. Further, nt position 1158 of *tuf*-1 from *B. abortus *strains 9–941 and 2308 was identical to those from *B. abortus *biovars 2 and 4 but differed from the other *B. abortus *biovars. Whereas a single nt varied among copies of *tuf*-1 and *tuf-*2 from *B. abortus *biovar 1 strain 9–941 and *B. suis *1330, eight nt varied between *B. suis *1330 and *B. melitensis *16 M. The other classical spp. and the marine *Brucella *were intermediate between *B. abortus*/*B. suis *and *B. melitensis *(Table 3).

### Erythromycin mutants

Ery^R ^mutants of several reference *Brucella *strains having MIC values less than 2 μg/ml and the three marine *Brucella *were selected. Mutant strains were not recovered from *B. ovis*, *B. abortus *biovar 2, or *B. suis *biovar 5. Three ribosomal associated loci, 23S rrn, *rplV*, and *rplD*, were analyzed from ery^R ^mutants of *B. suis *biovar 1, *B. canis*, *B. neotomae*, and the three marine *Brucella *(Table 4). The spontaneous ery^R ^rate among the classical *Brucella *spp. varied by 100-fold. Rates of mutation to ery^R ^and highest concentration of erythromycin allowing growth for each parental strains of the classical *Brucella *spp. were: *B. suis *3.7 × 10^-7 ^(5 μg/ml erythromycin); *B. canis *1.5 × 10^-8 ^(20 μg/ml erythromycin); and *B. neotomae *6.6 × 10^-8 ^(5 μg/ml erythromycin). All the marine ery^R ^isolates were selected from plates containing 20 μg/ml erythromycin, and mutational rates were 1.9 × 10^-6^, 5.1 × 10^-7^, and 2.7 × 10^-6 ^for porpoise, seal, and dolphin, respectively.

Though MIC values of the ery^R ^mutants of the classical strains *B. suis*, *B. canis*, and *B. neotomae *increased, they did not increase as much as those of the marine isolates (Table 4). While MIC values increased for the ery^R ^mutants of the classical *Brucella *strains, the increases were only 2 to 6-fold compared to 15 to > 256-fold for the marine *Brucella*. Over half of the marine ery^R ^mutants had erythromycin MIC values of 128 μg/ml or higher, and all the dolphin mutants had MIC values greater than 256 μg/ml. The clindamycin MIC values of the ery^R ^mutants were similar to those of the parental strains except for porpoise b which had a mutation in 23S *rrn *and the seal isolates a and c.

Mutations among the marine ery^R ^mutants were found in two ribosomal associated loci, 23S *rrn *and *rplD*. Only a single mutation was identified in 23S *rrn*. Porpoise isolate b had a mutation within the peptidyl transferase center of 23S *rrn*, nt 2058 (Ec), but all three 23S *rrn *copies were not mutated, as the signal was mixed. This mutant was resistant to both erythromycin and clindamycin, MIC values >256 μg/ml. Most ribosomal associated mutations occurred in *rplD *(Table 4), and these were only found among the marine isolates. The mutations were not random. Several ery^R ^isolates had mutations at nt 209 or nt 217. At nt 209, porpoise isolates a and d and dolphin isolates a and c had an A instead of a G, substituting an Asp for a Gly. Dolphin ery^R ^mutants isolates b and d had a T instead of a C at nt 217 of *rplD*, resulting in the incorporation a Cys of rather than an Arg. Seal ery^R ^isolate c had an A instead of a C at nt 217 which resulted in the incorporation of a Ser rather than an Arg. Two of the porpoise ery^R ^isolates c and e had deletions in *rplD*, resulting in the loss of 18 or 30 codons. The deletion of 18 aa in L4 of porpoise c is consistent with recombination between two copies of 5'GGG-CCG-CGC-3' occurring between nt 153–161 and 207–215.

Only one ribosomal associated loci mutation was identified among the *B. suis*, *B. canis*, and *B. neotomae *ery^R ^mutants by analyzes of 23S *rrn*, *rplD*, and *rplV*. A duplication of a six-bp repeat in the β-hairpin loop of L22 of *B. neotomae *isolate d expanded the number of Gly-Arg aa repeats from three to four (Fig. 2, Table 4). This mutant had a slightly higher MIC value for erythromycin.

### Efflux

Erythromycin and clindamycin MIC values of the reference strains and the ery^R ^mutants were analyzed in the presence of the efflux inhibitor PAβN (Tables 4 and 5). Using Etest strips, a decrease in MIC values could only be detected if the MIC values >256 μg/ml fell to or below 256 μg/ml. MIC values of the reference strains decreased variably in the presence of the inhibitor (Table 5). Though PAβN affected the erythromycin MIC value for *B. suis *biovar 1, reducing it two-fold or by two dilutions as per the Etest, the MIC values for the *B. suis *biovar 1 ery^R ^mutants were not affected. In the case of *B. abortus *biovar 5, even though its erythromycin MIC value was lowered in the presence of PAβN, the clindamycin MIC value was unaffected.

Efflux inhibition among the ery^R ^mutants by PAβN (Table 4) was variable among the strains. The *B. canis *and *B. neotomae *ery^R ^mutants had decreases in their erythromycin and clindamycin MIC values in the presence of PAβN. The seal ery^R ^mutants had increased erythromycin MIC values that were variably reduced in the presence of PAβN. For example, isolate a had an erythromycin MIC value of 24 μg/ml which was reduced to 12 μg/ml by PAβN, but isolate d had a MIC value of 128 μg/ml which was reduced to 16 μg/ml. Only the seal ery^R ^clindamycin MIC values were either identical to (>256 μg/ml) or lower than (96 and 16 μg/ml) that of the parental strain, and none were affected by PAβN. The dolphin ery^R ^mutants differed from all the other ery^R ^mutants in that they had uniform erythromycin MIC increases and the highest MIC increases of any of the other groups. Though all the dolphin ery^R ^mutants' erythromycin MIC values increased from 3 μg/ml to >256 μg/ml, their erythromycin MIC values were differentially affected by PAβN. In the presence of PAβN, two dolphin ery^R ^mutants had MIC values equal to or greater than 128 μg/ml while the rest had MIC values of 8 μg/ml or less. Like the porpoise isolates, except isolate b, all the dolphin isolates had lower clindamycin MIC values than the parental strain and the clindamycin MIC values were only slightly affected by PAβN.

### Phylogenetic tree

**Figure 4:**
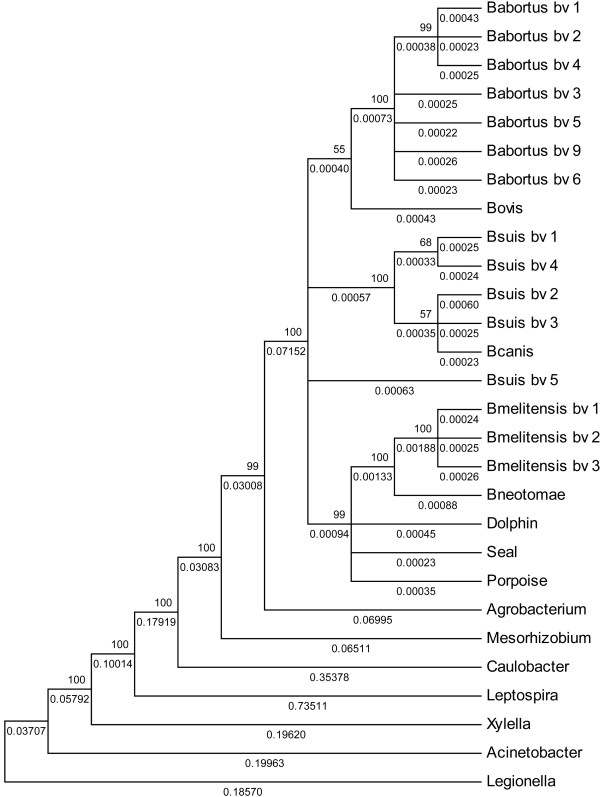
**Phylogeny of *Brucella *calculated using highly conserved ribosomal associated loci**. Shown is the single optimization alignment tree based on *rplV*, *tuf*-1, *tuf*-2, and 23S rrn sequences from 28 taxa consisting of the 21 *Brucella *strains (see Table 1), which included the 18 classical *Brucella *reference strains and three marine *Brucella*, and seven outgroups of known genomic sequences. Branch lengths (mean number of differences per residue along each branch) are given as well as bootstrap values (percentage of bootstrap support based on 100 replicates). *Legionella pneumophila *subspecies Pneumophila strain Philadelphia [GenBank:NC_002942] was used to root the tree. Other bacterial outgroups include: *Acinetobacter *species ADP1 [GenBank:NC_005966], *Caulobacter crescentus *CB15 [GenBank:NC_002696], *Leptospira interrogans *serovar Copenhagen strain Fiocruz L1-130 [GenBank:NC_005823], *Mesorhizobium loti *MAFF303099 [GenBank:BA000012], *Agrobacterium tumefaciens *C58 circular [GenBank:NC_003062] and linear chromosomes [GenBank:NC_003063], and *Xylella fastidosa *9a5c [GenBank:NC_002488].

A phylogenetic tree was constructed using concatenated 23S *rrn*, *rplV*, *tuf*-1, and *tuf*-2 (Fig. 4). *Brucella *formed a node with the closest clades being other α-*Proteobacteria*, *Agrobacterium, Mesorhizobium*, and *Caulobacter *followed by *Leptospira *and γ-*Proteobacteria*, *Xylella*, *Acinetobacter*, and the facultative intracellular animal pathogen *Legionella*. The cluster containing the *Brucella *species is robustly formed (high bootstrap values) into a distinct clade separate from the outgroups and forming four nodes subclustering: (1) *B. abortus *and *B. ovis*; (2) *B. suis *and *B. canis;*(3)*B. melitensis, B. neotomae *and the marine *Brucella*; and (4) *B. suis *biovar 5.

The tree constructed from a concatenated sequence, i.e. a supergene or supermatrix, was consistent with a concatenated tree calculated from individual loci (data not shown). Both trees supported classical classification, clustered the marine isolates with *B. melitensis*, and indicated intrinsic differences among marine *Brucella*. Bootstrap numbers (Fig. 4) were robust for all nodes (99 or 100) except *B. ovis*, which clustered with *B. abortus*; in the additive tree, *B. ovis *formed a unique branch. Though *B. suis *and *B. canis *composed a node, *B. suis *biovars 1 and 4 were on one branch and *B. suis *biovars 2 and 3 on another branch with *B. canis*. Shared 23S *rrn *polymorphisms divided the *Brucella *into three groups, placing *B. melitensis *between *B. abortus *and *B. suis *(Table 2). The *tuf*-1 and *tuf*-2 sequences (Table 3) separated *B. abortus *and *B. suis *and placed *B. neotomae *and the marine isolates intermediate between *B. abortus *and *B. suis *and *B. melitensis*. The *rplV *from *B. abortus *and *B. melitensis *were identical. Indels in *rplV *split *B. suis *biovars into two groups.

## Discussion

The Etest was used to determine MIC values of the classical reference *Brucella *spp., their biovars, and three marine isolates to macrolides and a lincosamide. Our results differed somewhat from those reported by Meyer [17] using antibiotic discs containing low or high concentrations of erythromycin. Meyer found *B. ovis *and *B. canis *more resistant than *B. suis *to erythromycin, but Etest MIC values for *B. ovis *and *B. canis *were less than those of the reference strains of *B. suis*. The MIC values for the marine isolates were low and more similar to those of *B. suis *than to those of either *B. melitensis *or *B. abortus*. The patterns of relative sensitivity to macrolides of the reference *Brucella *were similar for erythromycin, clarithromycin, and azithromycin but differed from that for clindamycin. The susceptibility of *B. suis *to relatively low concentrations of the macrolide azithromycin suggests that this antibiotic may be a beneficial treatment for *B. suis *infections as it has a long *in vivo *half-life (50 hours), concentrates in macrophages, and lacks uptake saturation [35].

Ribosomal associated loci 23S *rrn*, *rplD, rplV, tuf*-1, and *tuf*-2 were analyzed for polymorphisms. Three monomorphisms were identified among *rplD *loci, but only one of them resulted in a difference among the putative L4 sequences. Although polymorphism was high among the *tuf*-1 and *tuf*-2 loci, all were silent. Sequences among 23S *rrn *and *rpl*V loci were polymorphic.

The three polymorphic sites identified among the *Brucella *23S *rrn *loci separated them into three groups (Table 2). The only sequence difference among the 23S *rrn *peptidyl transferase centers of the reference *Brucella *strains was at nt 2610 (Ec), where there was either a T or a C. Many nucleotides in the peptidyl transferase center are conserved among bacteria and other organisms, but nt 2610 (Ec) is not. Either a T or C is common in bacteria. In any case, a T2610C (Ec) mutation in 23S *rrn *from *S. pneumonia *only slight affected its MIC values for macrolides and clindamycin [36]. Mutation of the peptidyl transferase center of 23S RNA (A2058G, Ec) of porpoise ery^R ^mutant isolate b increased the erythromycin and clindamycin MIC values from 1.5 and 16 μg/ml, respectively, to >256 μg/ml. These MIC values were unaffected by the presence of efflux inhibitor PAβN. Concurrent appearance of resistance to erythromycin and clindamycin by mutation of nt 2058 (Ec) is observed in other bacteria [23]. Methylation of either nt 2059 or 2058 (Ec) of the peptidyl transferase center reduces the sensitivities of bacteria to macrolides and lincosamides [26]. We were unable to identify homologs of any 23S *erm *methylation genes by BLAST [37], but, then, methylation of ribosomal rRNA is much more widely described in Gram-positive clinical isolates [26].

The *rpl*V sequences of the reference *Brucella *strains and marine *Brucella *were polymorphic, resulting in the differences among their putative L22 peptide sequences and lengths of the L22 β-hairpin loops. This was unexpected because L22 peptide sequence is conserved within a bacterial species [18,38] and the length of the L22 β-hairpin loop is highly conserved across biological kingdoms [38]. Differences in β-hairpin loop lengths among the *Brucella *L22 peptides were due to variable numbers of Gly-Arg repeats (Fig. 2). Though *B. neotomae *ery^R ^isolate d had four Gly-Arg repeats, due to a six base insertion, the mutant's erythromycin and clindamycin MIC values were only slightly increased.

The single amino acid difference found among the putative L4 sequences of the reference and marine strains could not be correlated with a difference in MIC values. Among the ery^R ^mutants, all but two of the mutations were identified in *rplD*, and, interestingly, they only occurred among the marine ery^R ^isolates. All erythromycin MIC values that increased among the ery^R ^marine isolates were lowered by the efflux inhibitor PAβN. Nevertheless, some of the MIC values remained relatively high in the presence of PAβN. The L4 peptides of these mutants may work in conjunction with or be dependent on specific efflux RND pumps as shown for *Haemophilus influenza *HMC-C [32,33].

The *tuf*-1 and *tuf*-2 loci were the most polymorphic of the ribosomal associated loci examined, yet their putative peptide sequences were identical. Strain sequence differences between *tuf*-1 and *tuf*-2 were confined to the borders. This is consistent with gene conversion occurring more efficiently within conserved sequences rather than near the borders. Given that *B. melitensis *and *B. abortus *genomes have fewer single nucleotide polymorphisms (SNP) between them than either has with *B. suis, tuf-1 and tuf-2 *from *B. abortus *and *B. melitensis *were expected to be highly similar. This was not the case. *Brucella abortus *and *B. suis tuf-1 *and *tuf*-2 had few sequence differences (Table 3). The *tuf*-1 and *tuf*-2 sequences from the marine isolates were intermediate between *B. abortus/B. suis *and *B. melitensis*/*B. neotomae*. The *tuf-1 and tuf-2 *genes encode a core metabolic product and the apparent selective pressure on conserving EF-Tu sequences in the face of *tuf*-1 and *tuf*-2 polymorphism supports different evolutionary paths [39] for *B. abortus *and *B. melitensis*.

MIC values and sequences of ribosomal related loci did not correlate with antibiotic susceptibility. To determine if efflux played a part in *Brucella *differential antibiotic resistance, we studied the effect of an RDF efflux inhibitor on MIC values. With the possible exception of *B. abortus *biovar 9, erythromycin MIC values of all the reference strains were reduced by the inhibitor PAβN though MIC values decreases were variable. Even low erythromycin MIC values decreased further in the presence of PAβN, demonstrating that efflux afforded the *Brucella *a low level of intrinsic antibiotic resistance similar to that reported for *Campylobacter *[40].

Many clinical isolates are resistant to antibiotics due to increased efflux as a result of mutations of efflux promoters and global and physically linked regulator genes or mobilization of insertion sequences (for a review see [28]). Most of the ery^R ^strains had increased antibiotic efflux, though the marine ery^R ^strains had larger increases in efflux than those of the classical reference strains of *B. suis *biovar 1, *B. canis*, and *B. neotomae *(Table 4). This suggests a fundamental biological difference between these groups. It is known that the marine *Brucella *have a high copy number [41] of the insertion sequence IS*711 *[42]. IS*711 *has been shown to mobilize in *Brucella *under stress or selective pressure [43,44] and could be a source of instability [42] in marine *Brucella*.

*Brucella *phylogenetic trees and dendrograms have been constructed based on genomics maps [3,5,6], amplified fragment length polymorphisms (AFLP) [45], multilocus enzyme electrophoresis (MLEE) [6], and outer membrane proteins *omp2a*/*omp2b *[3]. Now, other universally conserved loci, especially 23S rrn, EF-Tu, *rpo*B, and gyrase, are increasingly being used to establish relationships among highly similar bacteria with important phenotypic differences to determine their relationships [16]. We constructed a phylogenetic tree based on concatenated sequences of ribosomal associated loci. Most phylogenetic trees and dendrograms, including ours, place *B. abortus*, *B. suis*/*B. canis*, and *B. melitensis *on separate branches, supporting alternative evolutionary paths. Recently, it was shown that *Brucella *isolates could be identified at the species level using 21 variable number tandem repeats (VNTR) [46]. The neighbor joining tree based on VNTR data produced major clusters that encompassed the classical *Brucella *spp. On this tree, the reference *B. suis *biovar 5 strain, which appears as a unique branch on our tree, was shown to be only distantly related to all other reference strains and isolates by VNTR analyses [46]. Though *B. ovis *formed a single cluster by VNTR analyses, it clustered, albeit with a low bootstrap value, with *B. abortus *on our tree. Significant sequence differences have been reported between *B. ovis *and other classical *Brucella *spp. reference strains [47,48]. *Brucella neotomae *grouped with *B. melitensis *here but was on a separate node. Based on VNTR data, *B. neotomae *occurs on a unique branch but groups with *B. abortus *on a AFLP generated dendrogram [45]. Marine isolates are not found on many *Brucella *phylogenetic trees. Ours grouped the marine *Brucella *and *B. neotomae *with *B. melitensis *but on separate branches. This is in agreement with the genetic diversity observed among the marine isolates and proposals that marine isolates may comprise more than one species [3,41].

## Conclusion

Ribosomal associated polymorphisms among the reference *Brucella *spp. did not correlate with differential intrinsic antibiotic resistance to erythromycin or clindamycin. Efflux is an important mechanism of resistance to macrolides and the lincosamide clindamycin in *Brucella *and can be inhibited by the RND efflux inhibitor PAβN. A phylogenetic tree constructed based on concatenated ribosomal associated loci supports alternative evolutionary paths for *B. melitensis*, *B. abortus*, and *B. suis*, and clustered the marine *Brucella *with *B. melitensis*, and *B. canis *with *B. suis*. It also supports the doubtful close relationship of *B. suis *biovar 5 with *B. suis*.

## Methods

### Bacterial strains and growth conditions

Bacterial strains (Table 1) were obtained from our laboratory collection for this study. Bacteria were grown at 37°C on tryptose agar (DIFCO Laboratories, Detroit, MI) containing 5% bovine serum in the presence of 7.5% CO_2_. Cells were suspended in saline (10^10 ^CFU/ml), mixed with two volumes of methanol, and stored at 4°C until needed.

### Etest

*In vitro *activities of azithromycin, clarithromycin, erythromycin, and clindamycin were determined by the Etest (AB Biodisk, Piscataway, NJ). The highest MIC determination for these antibiotics using the Etest is 256 μg/ml. The preformed gradient of the Etest strips covers a continuous MIC range corresponding to 15 two-fold dilutions with a precision of 0.5 dilution. Bacterial inocula were prepared by adjusting the turbidity of a 48 h culture to a 0.5 McFarland standard (5 × 10^8 ^CFU/ml). The suspension was streaked onto Difco™ Mueller Hinton agar (Becton, Dickinson and Company, Sparks, MD) in the presence or absence of 25 μg/ml of efflux inhibitor PAβN [49] (Sigma Chemical Co., St. Louis, MO) using a cotton swab, and the Etest strips applied. Plates were incubated (37°C) in 7.5% CO_2_. Results were read after 48 h.

### Selection of erythromycin mutants

*Brucella *strains having erythromycin MIC values <5 μg/ml were suspended in saline and plated (10^8 ^cfu) in triplicate onto Difco™ Mueller Hinton agar containing 5, 10, or 20 μg/ml of erythromycin (Sigma Chemical Co.) and incubated at 37°C in the presence of CO_2_. Five colonies were selected from plates with the highest concentration of erythromycin supporting growth, and subsequently streaked onto tryptose serum agar and Difco™ Mueller Hinton agar containing erythromycin.

### PCR amplification

Master mixes for PCR reactions were prepared by use of the Fast Start Taq DNA polymerase kit (Roche Molecular Biochemicals, Indianapolis, IN) according to manufacturer's instructions. Methanol treated cells were diluted 1/10 in water and used immediately or stored at 4°C up to 2 months. One μL was added per 25 μL of reaction mixture. Reactions were 50 or 100 μL. Cells were disrupted and amplification initiated by heating the reactions to 95°C for 5 min. Melting, annealing, and elongation temperatures and times were 95°C for 15 sec, 60°C for 30 sec, and 72°C for 90 sec, respectively. After amplification for 35 or 40 cycles, elongation was extended by 4 min.

### Primers

The primer sets for amplification and sequencing of 23S *rrn *genes annealed to *rrn*A from *B. abortus *9–941 [GenBank:AE17223, BruAb1_rrna_0005]: nt 37–62 (CAT-GCA-CAG-GCG-ATG-AAG-GAC-GTG-AT) and nt 540–518 (GGA-TTT-CAC-GTG-TCC-CGC-CCT-ACT-CA) (note that this set amplified intervening sequence); nt 455–481 (AGT-TGG-AAA-ACT-CGA-CCG-AAG-TGG-GTG) and nt 1153-1127 (CCT-TAG-ATG-GTG-GTC-AGG-GTT-GTT-GCC); nt 1013–1039 (GAG-CAC-TGG-ATG-GGC-TAT-GGG-GAC-TCA) and nt 1732-1707 (GTG-CAT-TTT-GCC-GAG-TTC-CTT-CAA-CG); nt 1868–1894 (CCG-GTG-CTG-GAA-GGT-TAA-GAG-GAG-AGG) and nt 2605-2579 (CCC-AAC-TCA-CGT-ACC-GCT-TTA-AAT-GGC); and nt 2505–2529 (CGG-GGT-TGT-TTG-GCA-CCT-CGA-TAT-C) and nt 2850-2825 (CCC-GGC-CTA-TCA-ACG-TGG-TGG-TCT-TC). Primers used in PCR reactions to amplify the 3' end based on the genomic sequence of *rrn*C from *B. suis *[GenBank:NC_004311, Bs23SC] were forward (GGT-TTC-CCG-CTT-AGA-TGC-CTT-CAG-GA) and reverse 1 (CTT-CAG-AGA-TTA-TCC-CGT-CCG-TAT-ATA-TCT-ACC) and reverse 2 (ATA-GTG-ATC-CGG-TGG-TCC-CGC-GTG). Primers based on 23S *B. suis rrn*C unique sequences and *B. suis *sequences flanking *rrn*C, respectively, were: (GGG-TCC-AGG-ACC-GTG-TAT-GGT-GGG-TAG) and (CTT-CCA-TCC-ATG-AGC-GGC-AAA-GGA-AAT-G). Primers for amplification of L4, L22, EF-Tu1, and EF-Tu2 were: L4 forward 1, (ACG-ACC-ACG-ATC-TGC-CGA-AGA-AGG-TTC), and reverse, (GCC-ACG-TTG-AAG-ACG-ACC-TGG-TG); L4 forward 2, (GTG-TTC-AAG-GGC-AAG-AAG-ATG-GCT-GGT-C) and reverse 2, (GAT-CTC-CGC-ACC-GCC-GAT-AAG-AAG-TG); L22 forward, (GCG-GAT-CTT-GAC-ATC-TTC-ATG-CAG-CAG), and reverse, (TTG-TCG-GTC-TGA-CTT-TCG-GCG-TCT-ACA); EF-Tu1, forward, (TCA-AGG-CGA-ATG-CGG-ATG-TTT-TGA-CC) and reverse (GCG-GTC-GCA-CAG-GAA-ATC-CAG-AAG-AAG); and EF-Tu2 forward, (GCG-GGG-AAT-TAT-CTC-GGC-AGC-ACT), and reverse, (CGA-GCG-GTA-TGG-CGT-GTA-AGG-AAT-CAT). Primers internal to the PCR products were synthesized as necessary to obtain sequences of the products.

### Sequence determination and comparisons

Amplified products were purified (QIAquick PCR Purification Kit, Qiagen, Valencia, CA) and sequenced at the Genomics Center at the National Animal Disease Center, Ames, IA (ABI Prism 3700 DNA Analyzer) using primers as listed above. For large products, internal primers were synthesized as needed to obtain coding sequences. Sequences were assembled and aligned, and polymorphisms were identified by use of Sequencer 3.1.2 (Genes Codes Corp., Ann Arbor, MI). In some cases, MacVector (ClustalW) was used for sequence comparisons.

### Protein folding

The Swiss-Pdb viewer software version 3.7 [50,51] was used to predict folding of L22 based on the coordinates determined for L22 from *Thermus thermophilus *[52].

### 23S rrn base numbering

The numbering for *Brucella *23S *rrn *is based on *rrn*A from *B. abortus *9–941 [GenBank:NC_006932, Gene ID: 3339965]. When the nts refer to *E. coli *23S rRNA *rrn*G [GenBank:U00096, GI:48994873], the nts are followed by (Ec).

### Accession numbers

Genome and genomic sequences referred to in this study: *B. suis *1330 [GenBank:AE014291 and AE014292], *B. melitensis *16 M [GenBank:AE008917 and AE008918], *B. abortus *9–941 [GenBank:AE017223 and AE017224], *B. abortus *2308 [GenBank:AM040264], and *B. abortus rrn*A sequence [GenBank:NC_006932, Gene ID: 3339965]. Genomic sequences determined in this study: L4 (*rplD*) [GenBank:DQ289557 to DQ289577], L22 (*rplV*) [GenBank:DQ227901 to DQ227921], EF-Tu1 (*tuf*-1) [GenBank:DQ227922 to DQ227942], EF-Tu2 (*tuf*-2) [DQ227943 to DQ227963], 23S *rrn *region 1 (nt 69 to 1678) [GenBank:DQ287886–DQ287906], and 23S *rrn *region 2 (nt 1920–2807) [GenBank:DQ287865–DQ287885]. Ery^R ^mutant strain ribosomal associated sequences: *B. neotomae *(*rplV*) [GenBank:DQ659536], porpoise b (23S *rrn*) [GenBank:DQ659537], dolphin a-d (*rplD*) [GenBank:DQ660399–DQ660402], porpoise a, c-e (*rplD*) [GenBank:DQ6600403–DQ6600406], and seal c (*rplD*) [GenBank:DQ660407].

### Dendrogram

Data sets consisted of concatenated genes (*rplV*, *tuf*-1, *tuf*-2) and sequences from 23S *rrn *from 28 taxa consisting of the 18 *Brucella *reference strains, three marine isolates, and seven outgroups of known genomic sequences (see Table 1 and Fig. 4). 23S *rrn *intervening sequences were eliminated from the comparison. Assembled sequences were aligned using ClustalW v1.83 [53]. Each nucleotide data set was then analyzed under the optimal criteria of maximum likelihood using MrBayes v3.1.2 Baysian analysis and Markov Chain Monte Carlo methods to search tree space and infer posterior distribution of topologies [54]. Settings for MrBayes were the general time reversible substitution model with sites drawn from a gamma distribution. The outgroup for MrBayes was *Legionella pneumophila *subspecies Pneumophila strain Philadelphia [GenBank:NC_002942]. The number of generations was set at 1,000,000, number of chains at 4, print frequencies at 10,000, and sample frequencies at 100. Branch lengths were saved and all other settings were default. The evolutionary tree was displayed using Tree Explorer [55] based on options used to compute or display the phylogeny.

## Abbreviations

Standard three letter code was used for amino acids and standard one letter code for DNA bases.

## Authors' contributions

SH identified the loci for study; designed primers, amplified loci, prepared templates for sequencing, analyzed sequence data and drafted the manuscript. AJ cultured the *Brucella*, determined MIC values, prepared L22 predicted structures, and edited the manuscript. SH and AJ prepared graphs, tables, dendograms, and analyzed data. Both authors have read the manuscript and approved the final version.

## Figures and Tables

**Table 1:** Strains of *Brucella *used in this study.

**Species**	**Biovar**	**Strain**	**Host**	**Origin**	**Reference**
*B. abortus*	1	544	Cattle	England	ATCC* 23448
	1	9–941	Cattle	USA	11
	2	86/8/59	Cattle	England	ATCC 23449
	3	Tulya	Cattle**	Uganda	ATCC 23450
	4	292	Cattle	England	ATCC 23451
	5	B3196	Cattle	England	ATCC 23452
	6	870	Cattle	Africa	ATCC 23453
	9	C68	Cattle	England	ATCC 23455
*B. canis*		RM6/66	Dog	USA	ATCC 23365
*B. melitensis*	1	16 M	Goat	USA	ATCC 23456
	2	63/9	Goat	Turkey	ATCC 23457
	3	Ether	Goat**	Italy	ATCC 23458
*B. neotomae*		5K33	Wood rat	USA	ATCC 23459
*B. ovis*		63/290	Sheep	Africa	ATCC 25840
*B. suis*	1	1330	Pig	USA	ATCC 23444
	2	Thomsen	Hare	Denmark	ATCC 23445
	3	686	Pig**	USA	ATCC 23446
	4	40	Reindeer	USSR	ATCC 23447
	5	513	Mouse	USSR	***
*Brucella *spp.		2/94	Seal	Scotland	6
("*maris*")		1/94	Porpoise	Scotland	6
		14/94	Dolphin	Scotland	6

*ATCC, American Type Culture Collection, Beltsville, MD; **Isolate from human; ***Reference strain 513 not deposited in ATCC.

**Table 2:** *Brucella *23S *rrn *polymorphisms^&^.

***^#^Brucella *strains**	*Nt 1085 934 (Ec)		*Nt 1564 1423 (Ec)		*Nt 2632 2610 (Ec)	
			
	**A**	**G**	**A**	**G**	**T**	**C**
Ba b1		X		X		X
Ba b2		X		X		X
Ba b3		X		X		X
Ba b4		X		X		X
Ba b5		X		X		X
Ba b6		X		X		X
Ba b9		X		X		X
Bc	X		X		X	
Bs b1	X		X		X	
Bs b2	X		X		X	
Bs b3	X		X		X	
Bs b4	X		X		X	
Bs b5		X	X			X
Bo		X	X			X
Bn		X	X			X
Bm b1		X	X			X
Bm b2		X	X			X
Bm b3		X	X			X
Dolphin		X	X			X
Porpoise		X	X			X
Seal		X	X			X

^&^GenBank accession numbers for each *Brucella *23S *rrn *sequences are listed in Methods. ^#^*Brucella *strains are as listed in Table 1. *Nt positions as per *B. abortus *23S *rrn*A [GenBank:AE017223, BruAb1_rrna_0005, Gene ID: 3339965]. (Ec) denotes position in *Escherichia coli *23S *rrn *[GenBank:U00096].

**Table 3:** Polymorphic sites among *tuf*-1 and *tuf*-2 from *Brucella*.

^$^**Nt position**	**12**		**36**		**141**		**183**		**198**		**219**		**345**		**378**		**511**		**609**		**936**		**1158**	
	
^#^**Bru↓|Nt→**	**T**	**C**	**C**	**T**	**C**	**T**	**C**	**A**	**G**	**A**	**A**	**G**	**C**	**T**	**C**	**T**	**C**	**A**	**G**	**A**	**C**	**T**	**C**	**T**
Bab 9–941	X		X		X		X		X		X		X		X		X		X		X		**2**	**1**
Bab b1	X		X		X		X		X		X		X		X		X		X		X			X
Bab b2	X		X		X		X		X		X		X		X		X		X		X		**2**	**1**
Bab b3	X		X		X		X		X		X		X		X		X		X		X		X	
Bab b4	X		X		X		X		X		X		X		X		X		X		X		**2**	**1**
Bab b5	X		X		X		X		X		X		X		X		X		X		X		X	
Bab b6	X		X		X		X		X		X		X		X		X		X		X		X	
Bab b9	X		X		X		X		X		X		X		X		X		X		X		X	

B. suis b1	X		X		X		X		X		X		X		X		X		X		X		X	
B. suis b2	**1**	**2**	X		X		X		X		X		X		X			**X**	X		X		X	
B. suis b3	**1**	**2**	X		X		X		X		X		X		X		X		X		X		X	
B. suis b4	X		X		X		X		X		X		X		X		X		X		X		X	
B. suis b5	**1**	**2**	X		X		X		X		X		X		X		X		X		X		X	

Bmel b1	X			**X**		**X**		**X**		**X**		**X**		**X**		**X**	X		X			**X**	X	
Bmel b2	X			**X**		**X**		**X**		**X**		**X**		**X**		**X**	X		X			**X**	X	
Bmel b3	X			**X**		**X**		**X**		**X**		**X**		**X**		**X**	X		X			**X**	X	

Bneo		**X**	X			**X**		**X**		**X**		**X**	X			**X**	X			**X**	X		X	
B. ovis	X		X		X		X		X		X		X		X		X		X		X		X	
B. canis	**1**	**2**	X		X		X		X		X		X		X		X		X		X		X	

Seal	**1**	**2**	X		X		X		X			**X**	X			**X**	X		X		X		X	
Dolphin	**1**	**2**	X		X		X		X			**X**	X			**X**	X		X		X		X	
Porpoise	X		X		X		X		X			**X**	X			**X**	X		X		X		X	

^$^Nt = nucleotide; ^#^*Brucella *strains, see Table 1; X = *tuf*-1 and *tuf*-2 have identical sequences; 1 = *tuf*-1 sequence only, 2 = *tuf*-2 sequence only. Polymorphic sites are in **bold**. *tuf*-1 and *tuf*-2 GenBank accession numbers are listed in Materials and methods.

**Table 4:** Determination and characterization of erythromycin and clindamycin MICs and molecular characterization of *Brucella *ery^R ^mutants.

***Brucella ery***^**R **^ ***strains***		**ERY**	**ERY + PAβN**	**CL**	**CL + PAβN**	**Loci**	**Mutation**
*B. suis*	WT	1.5	0.75	8	4	NA	
	a	6	6	12	12	-	
	b	6	6	12	12	ND	
	c	4	6	8	8	-	
	d	6	6	12	12	ND	
	e	6	6	8	8	ND	

*B. canis*	WT	0.5	0.094	8	0.5	NA	
	a	2	0.32	8	1.0	-	
	b	2	0.047	6	<0.016	-	
	c	3	0.047	8	0.38	-	
	d	3	0.047	4	0.125	-	
	e	2	0.19	8	3	-	

*B. neotomae*	WT	0.75	0.047	3	0.75	NA	
	a	4	0.125	8	0.75	-	
	b	3	0.125	6	2	ND	
	c	2	0.064	6	1.5	-	
	d	6	0.25	6	1.0	L22	6 bp indel, β-loop
	e	2	0.38	4	2	-	

Propoise	WT	1.5	0.25	16	3	NA	
	a	16	0.75	4	3	L4	G209A; Gly70Asp
	b	>256	>256	>256	>256	23S	A2058G (Ec)
	c	192	1.5	4	3	L4	Δ18aa (54–71)
	d	48	0.38	6	3	L4	G209A; Gly70Asp
	e	128	1.5	4	3	L4	Δ30aa (54–81)

Seal	WT	6	1.5	>256	48	NA	
	a	24	12	96	96	-	
	b	32	24	>256	>256	-	
	c	>256	>256	16	16	L4	C217A; Arg73Ser
	d	128	16	>256	>256	-	
	e	128	24	>256	>256	ND	

Dolphin	WT	3	0.38	2	0.75	NA	
	a	>256	128	1.0	1.5	L4	G209A; Gly70Asp
	b	>256	6	1.5	1.0	L4	C217T; Arg73Cys
	c	>256	192	1.5	1.5	L4	G209A; Gly70Asp
	d	>256	8	2.0	1.0	L4	C217T; Arg73Cys
	e	>256	8	1.5	1.0	ND	

Mutants examined were isolates of *B. suis *biovar 1*, B. canis, B. neotomae*, and marine porpoise, seal and dolphin strains as per Table 1; WT, wild type; Ery, erythromycin; CL, clindamycin, PAβN, efflux inhibitor L-phenylalanine-L-arginine-β-naphthylamide; -, no mutation identified in any of three ribosomal loci examined; NA, not applicable; ND, sequence not determined.

**Table 5:** Effect of efflux on MICs *of Brucella *reference strains for erythromycin and clindamycin.

***Brucella***	**MIC**			
	
	**Ery**	**Ery + PAβN**	**CL**	**CL + PAβN**
B. *ab *b1	>256	96	>256	96
B. *ab *b2	0.19	0.0275	3	0.25
B. *ab *b3	128	64	192	128
B. *ab *b4	128	64	192	64
B. *ab *b5	>256	192	128	128
B. *ab *b6	>256	256	>256	96
B. *ab *b9	>256	>256	>256	>256
B. *mel *b1	16	4	24	16
B. *mel *b2	>256	48	64	12
B. *mel *b3	256	48	64	32
*B. suis *b1	1.5	0.75	8	4
*B. suis *b2	0.125	0.032	3	0.047
*B. suis *b3	1.5	1.0	24	48
*B. suis *b4	2	1.5	24	32
*B. suis *b5	0.094	0.047	6	2
*B. canis*	0.50	0.094	8	0.50
*B. ovis*	0.064	<0.016	3	<0.016
B. *neo*	0.75	0.047	3	0.75
Porpoise	1.5	0.25	16	3
Seal	6	1.5	>256	48
Dolphin	3	0.38	2	0.75

*Brucella *strains are as listed in Table 1; MIC, minimum inhibitory concentration; Ery, erythromycin; CL, clindamycin; PAβN, efflux inhibitor L-phenylalanine-L-arginine β-naphthylamide; b, biovar; ab, *abortus*; *mel*, *melitensis*; *neo*, *neotomae*.

## References

[B1] Meyer ME (1964). The epizootiology of brucellosis and its relationship to the identification of *Brucella* organisms. Am J Vet Res.

[B2] Meyer ME (1966). Host-parasite relationships in brucellosis I. reservoirs of infection and interhost transmissibility of the parasite. 70th Proc Annu Meet U S Livestock Sanit Assoc.

[B3] Cloeckaert A, Verger JM, Grayon M, Paquet JY, Garin-Bastuji B, Foster G, Godfroid J (2001). Classification of *Brucella* spp. isolated from marine mammals by DNA polymorphism at the *omp2* locus. Microbes Infect.

[B4] Alton GG, Jones LM, Angus RD, Verger JM (1988). Techniques for the Brucellosis Laboratory.

[B5] Michaux-Charachon S, Bourg G, Jumas-Bilak E, Guigue-Talet P, Allardet- Servent A, O'Callaghan D, Ramuz M (1997). Genome structure and phylogeny in the genus *Brucella*. J Bacteriol.

[B6] Gandara B, Merino AL, Rogel MA, Martinez-Romero E (2001). Limited genetic diversity of *Brucella* spp.. J Clin Microbiol.

[B7] Jahans KL, Foster G, Broughton ES (1997). The characterisation of *Brucella* strains isolated from marine mammals. Vet Microbiol.

[B8] Verger JM, Grimont F, Grimont PAD, Grayon M (1985). *Brucella*, a monospecific genus as shown by deoxyribonuclease acid hybridization. Int J Syst Bacteriol.

[B9] Woese CR, Fox GE (1977). Phylogenetic structure of the prokaryotic domain: the primary kingdoms. Proc Natl Acad Sci USA.

[B10] Moreno E, Cloeckaert A, Moriyon I (2002). *Brucella* evolution and taxonomy. Vet Microbiol.

[B11] Halling SM, Peterson-Burch BD, Bricker BJ, Zuerner RL, Qing Z, Li LL, Kapur V, Alt DP, Olsen SC (2005). Completion of the genome sequence of *Brucella abortus* and comparison to the highly similar genomes of *Brucella melitensis* and *Brucella suis*. J Bacteriol.

[B12] Gee JE, De BK, Levett PN, Whitney AM, Novak RT, Popovic T (2004). Use of 16S rRNA gene sequencing for rapid confirmatory identification of *Brucella* isolates. J Clin Microbiol.

[B13] DelVecchio VG, Kapatral V, Redkar RJ, Patra G, Mujer C, Los T, Ivanova N, Anderson I, Bhattacharyya A, Lykidis A, Reznik G, Jablonski L, Larsen N, D'Souza M, Bernal A, Mazur M, Goltsman E, Selkov E, Elzer PH, Hagius S, O'Callaghan D, Letesson JJ, Haselkorn R, Kyrpides N, Overbeek R (2002). The genome sequence of the facultative intracellular pathogen *Brucella melitensis*. Proc Natl Acad Sci USA.

[B14] Paulsen IT, Seshadri R, Nelson KE, Eisen JA, Heidelberg JF, Read TD, Dodson RJ, Umayam L, Brinkac LM, Beanan MJ, Daugherty SC, Deboy RT, Durkin AS, Kolonay JF, Madupu R, Nelson WC, Ayodeji B, Kraul M, Shetty J, Malek J, Van Aken SE, Riedmuller S, Tettelin H, Gill SR, White O, Salzberg SL, Hoover DL, Lindler LE, Halling SM, Boyle SM, Fraser CM (2002). The *Brucella suis* genome reveals fundamental similarities between animal and plant pathogens and symbionts. Proc Natl Acad Sci USA.

[B15] Dewhirst FE, Shen Z, Scimeca MS, Stokes LN, Boumenna T, Chen T, Paster BJ, Fox JG (2005). Discordant 16S and 23S rRNA gene phylogenies for the genus *Helicobacter*: implications for phylogenetic inference and systematics. J Bacteriol.

[B16] vanWaasbergen LG, Miller RV, Day MJ (2004). What makes a bacterial species: when molecular sequence data are used is rRNA enough?. Microbial Evolution: Gene Establishment, Survival, and Exhange.

[B17] Meyer M, Adams LG (1990). Evolutionary development and taxonomy of the genus *Brucella*. Advances in Brucellosis Research.

[B18] Berisio R, Schluenzen F, Harms J, Bashan A, Yonath A (2003). Structural insight into the role of the ribosomal tunnel in cell regulation. Nat Struct Biol.

[B19] Nissen P, Hansen J, Ban N, Moore PB, Steitz TA (2000). The structural basis of ribosome activity in peptide bond synthesis. Science.

[B20] Tenson T, Lovmar M, Ehrenberg M (2003). The mechanism of action of macrolides, lincosamides and streptogramin B reveals the nascent peptide exit path in the ribosome. J Mol Biol.

[B21] Schlunzen F, Zarivach R, Harms J, Bashan A, Tocilj A, Albrecht R, Yonath A, Franceschi F (2001). Structural basis for the interaction of antibiotics with the peptidyl transferase centre in eubacteria. Nature.

[B22] Vester B, Douthwaite S (2001). Macrolide resistance conferred by base substitutions in 23S rRNA. Antimicrob Agents Chemother.

[B23] Tu D, Blaha G, Moore PB, Steitz TA (2005). Structures of MLSBK antibiotics bound to mutated large ribosomal subunits provide a structural explanation for resistance. Cell.

[B24] Amit M, Berisio R, Baram D, Harms J, Bashan A, Yonath A (2005). A crevice adjoining the ribosome tunnel: hints for cotranslational folding. FEBS Lett.

[B25] Chittum HS, Champney WS (1994). Ribosomal protein gene sequence changes in erythromycin-resistant mutants *Escherichia coli*. J Bacteriol.

[B26] Roberts MC, Sutcliffe J, Courvalin P, Jensen LB, Rood J, Seppala H (1999). Nomenclature for macrolide and macrolide-lincosamide-streptogramin B resistance determinants. Antimicrob Agents Chemother.

[B27] Zgurskaya HI, Nikaido H (2000). Multidrug resistance mechanisms: drug efflux across two membranes. Mol Microbiol.

[B28] Piddock LJV (2006). Clinically relevant chromosomally encoded multidrug resistance efflux pumps in bacteria. Clin Microbiol Rev.

[B29] Hogg T, Mesters JR, Hilgenfeld R (2002). Inhibitory mechanisms of antibiotics targeting elongation factor Tu. Curr Protein Pept Sci.

[B30] Poole K (2002). Outer membranes and efflux: the path to multidrug resistance in Gram-negative bacteria. Curr Pharm Biotechnol.

[B31] Nishino K, Latifi T, Groisman EA (2006). Virulence and drug resistance roles of multidrug efflux systems of *Salmonella enterica* serovar Typhimurium. Mol Microbiol.

[B32] Clark C, Bozdogan B, Peric M, Dewasse B, Jacobs MR, Appelbaum PC (2002). In vitro selection of resistance in *Haemophilus influenzae* by amoxicillin-clavulanate, cefpodoxime, cefprozil, azithromycin, and clarithromycin. Antimicrob Agents Chemother.

[B33] Peric M, Bozdogan B, Galderisi C, Krissinger D, Rager T, Appelbaum PC (2004). Inability of L22 ribosomal protein alteration to increase macrolide MICs in the absence of efflux mechanism in *Haemophilus influenzae* HMC-S. J Antimicrob Chemother.

[B34] Bricker BJ (2000). Characterization of the three ribosomal RNA operons *rrn*A, *rrn*B, and *rrn*C, from *Brucella melitensis*. Gene.

[B35] Gordon ME, Blumer JL (2004). Rationale for single and high dose treatment regimens with azithromycin. Pediatr Infect Dis J.

[B36] Garza-Ramos G, Xiong L, Zhong P, Mankin A (2001). Binding site of macrolide antibiotics on the ribosome: new resistance mutation identifies a specific interaction of ketolides with rRNA. J Bacteriol.

[B37] Altschul SF, Gish W, Miller W, Meyers EW, Lipman DJ (1990). Basic local alignment search tool. J Mol Biol.

[B38] Bowers AK, Keller JA, Dutcher SK (2003). Molecular markers for rapidly identifying candidate genes in *Chlamydomonas reinhardtii*: ERY1 and ERY2 encode chloroplast ribosomal proteins. Genetics.

[B39] Moreno E, Stackebrandt E, Dorsch M, Wolters J, Busch M, Mayer H (1990). *Brucella abortus* 16S rRNA and lipid A reveal a phylogenetic relationship with members of the alpha-2 subdivision of the class *Proteobacteria*. J Bacteriol.

[B40] Corcoran D, Quinn T, Cotter L, Fanning S (2006). An investigation of the molecular mechanisms contributing to high-level erythromycin resistance in *Campylobacter*. Int J Antimicrob Agents.

[B41] Bricker BJ, Ewalt DR, MacMillan AP, Foster G, Brew S (2000). Molecular characterization of *Brucella* strains isolated from marine mammals. J Clin Microbiol.

[B42] Halling SM, Tatum FM, Bricker BJ (1993). Sequence and characterization of an insertion sequence, IS*711*, from *Brucella ovis*. Gene.

[B43] Schurig GG, Roop RM, Bagchi T, Boyle S, Buhrman D, Sriranganathan N (1991). Biological properties of RB51; a stable rough strain of *Brucella abortus*.. Vet Microbiol.

[B44] Vemulapalli R, McQuiston JR, Schurig GG, Sriranganathan N, Halling SM, Boyle SM (1999). Identification of an IS*711* element Interrupting the *wbo*A gene of *Brucella abortus* vaccine strain RB51 and a PCR assay to distinguish strain RB51 from other *Brucella* species and strains. Clin Diagn Lab Immunol.

[B45] Whatmore AM, Murphy TJ, Shankster S, Young E, Cutler SJ, Macmillan AP (2005). Use of amplified fragment length polymorphism to identify and type *Brucella* isolates of medical and veterinary interest. J Clin Microbiol.

[B46] Whatmore AM, Shankster SJ, Perrett LL, Murphy TJ, Brew SD, Thirlwall RE, Cutler SJ, MacMillan AP (2006). Identification and characterization of variable-number tandem-repeat markers for typing of *Brucella* spp.. J Clin Microbiol.

[B47] Ficht TA, Bearden SW, Sowa BA, Marquis H (1990). Genetic variation at the *omp2* porin locus of the brucellae: species-specific markers. Mol Microbiol.

[B48] Rajashekara G, Glasner JD, Glover DA, Splitter GA (2004). Comparative whole-genome hybridization reveals genomic islands in *Brucella* species. J Bacteriol.

[B49] Lomovskaya O, Warren MS, Lee A, Galazzo J, Fronko R, Lee M, Blais J, Cho D, Chamberland S, Renau T, Leger R, Hecker S, Watkins W, Hoshino K, Ishida H, Lee VJ (2001). Identification and characterization of inhibitors of multidrug resistance efflux pumps in *Pseudomonas aeruginosa*: novel agents for combination therapy. Antimicrob Agents Chemother.

[B50] Guex N, Peitsch MC (1997). SWISS-MODEL and the Swiss-Pdb Viewer: an environment for comparative protein modeling. Electrophoresis.

[B51] DeepView Swiss-Pdb Viewer. http://www.expasy.org/spdbv.

[B52] Unge J, Berg A, Al-Kharadaghi S, Nikulin A, Nikonov S, Davydova N, Nevskaya N, Garber M, Liljas A (1998). The crystal structure of ribosomal protein L22 from *Thermus thermophilus*: insights into the mechanism of erythromycin resistance. Structure.

[B53] Thompson JD, D G Higgins DG, Gibson TJ (1994). CLUSTAL W: improving the sensitivity of progressive multiple sequence alignment through sequence weighting, position-specific gap penalties and weight matrix choice.. Nucleic Acids Res.

[B54] Huelsenbeck JP, Ronquist F, Nielsen R, Bollback JP (2001). Bayesian inference of phylogeny and its impact on evolutionary biology. Science.

[B55] Kumar S, Tamura K, Nei M (2004). MEGA3: integrated software for molecular evolutionary genetics analysis and sequence alignment. Brief Bioinform.

